# Mechanical Behaviour of Silicone Membranes Saturated with Short Strand, Loose Polyester Fibres for Prosthetic and Rehabilitative Surrogate Skin Applications

**DOI:** 10.3390/ma12223647

**Published:** 2019-11-06

**Authors:** Richard Arm, Arash Shahidi, Tilak Dias

**Affiliations:** Advanced Textiles Research Group, Flexural Composites Research Laboratory, School of Art and Design, Nottingham Trent University, Nottingham NG1 4GG, UK; arash.shahidi@ntu.ac.uk (A.S.); tilak.dias@ntu.ac.uk (T.D.)

**Keywords:** PDMS, silicone, fibre reinforced elastomers, prosthetic design, maxillofacial prosthesis, skin simulant

## Abstract

Silicone-based elastomers saturated with embedded, short-strand fibres are used for their ability to mimic the aesthetic qualities of skin in clinical and theatrical maxillofacial appliance design. Well-known to prostheses fabricators and technicians, the mechanical impact of fibre addition on elastomeric behaviour endures as tacit, embodied knowledge of the craft, almost unknown in the literature. To examine mechanical changes caused by fibre addition, 100 modified polydimethylsiloxane (PDMS) elastomeric compounds containing incremental amounts of loose polyester fibres were prepared and examined in a variety of mechanical tests. It was found that elasticity and strain percentage at breaking point was reduced by increasing fibre content, but Young’s modulus and ultimate tensile strength (UTS) increased. As fibre content was increased, strain hardening was seen at low strain rates, but exaggerated plastic deformation at high strain rates. PDMS hardness increased by 5 degrees of hardness (Shore-00 scale) for every additional percentage of fibres added and a strong positive linear coefficient (0.993 and 0.995) was identified to reach the hardness values given in the literature for living human skin. The apparent reorienting of loose fibres in the PDMS interrupts and absorbs stress during the loading process similar to the organic response to soft tissue loading, except in extension.

## 1. Introduction

Elastomers saturated with embedded, loose, short-strand fibres (flock) are known in clinical anaplastology and theatrical prosthesis appliance design for their ability to mimic the aesthetic qualities of skin. Fibres such as polyester, rayon or polyamide are produced in various lengths and colours and blended with a translucent liquid, poly-di-methyl-siloxane elastomeric gel (PDMS) to reflect the translucency, intrinsic pigmentation and texture of the living equivalent. Although well-known in the prosthesis industry for decades, currently, this knowledge is mainly tacit, embodied knowledge of the fabricators and technicians, and the impact these fibres have on the behavioural characteristics of soft PDMS elastomers remains almost unknown in the literature.

A limited study on stiffer PDMS and other elastomers (>20 A shore hardness) with embedded short-strand rayon fibres was reported by Sreeja [1] and Abdulla [2], but essential quantitative data on the relationship between fibre quantity and the mechanical properties of PDMS was not reported. The aesthetic value of short-strand fibre flock saturation in PDMS elastomeric gels was noted by Debreceni [3] and Montgomery [4], but they focused on aesthetic aspects and frequency of use in maxillofacial and theatrical prosthesis fabrication, lacking data on the behavioural implications or explicit, repeatable production methodology.

Fibre reinforced, medical grade, flexible composites (ethylene-propylene-diene-monomer) used in the manufacture of medical devices such as vascular stents, heart valves cardiac assistive devices, surgical grafts and sutures draw their biomechanical design inspiration from the arrangement and function of natural fibres such collagen and elastin, that are found in their organic counterparts [5,6]. Drawing inspiration from living fibrous membranes such as blood vessels and valves, such devices distribute and dissipate mechanical loading of the elastomers with complimentary, reinforcing fibres that have been embedded within the elastomeric lamina structure [7]. In dermatology, it is known that the amount of collagen and elastin found in the skin effects the characteristic performance and can be reliably measured with regards to hardness, deformation and strain-three key mechanical characteristics to consider when designing artificial skin for theatrical and maxillofacial prosthesis [7,8,9,10,11].

Soft, synthetic elastomers (lacking fibre reinforcement), such as PDMS gels, have an exaggerated isotropic viscoelastic elongation under load (up to 1000%) whereas soft, biological elastomers tissues like human skin (that contain fibres) exhibit less elongation under load (up to 150%).

It is tempting to argue then, that by varying the amount and architecture of embedded fibres in liquid PDMS we can gain control of mechanical behaviour, limit extensibility and improve strength by redistributing mechanical loading and interrupting the propagation of tears [5].

Recent studies into the suitable percentage of fibre saturation in other elastomers show that low volumes (by weight) of 2% to 2.5% introduce changes in mechanical behaviour, particularly in hardness and elastic modulus. [9,10] However, emulating the characteristics of living skin with fibre filled elastomers cannot be limited to mimicking simple hardness and isotropic, elastic modulus. Multi-axial deformation is inherent to the tactility of living flesh and cannot be ignored when mimicking the viscoelastic response of living skin. Heterogenic by nature, skins’ variable thickness and viscoelastic, time-dependent response to strain changes are dependent on age, gender, sample site and many other variables including fibre type, density and distribution. These variables are further confounded by the presence of incompressible fluids, hydrated or perfused tissue, embedded vascular structures and the volume, distribution and orientation of fibres such as collagen and elastin throughout each layer, each with their specific mechanical traits and contributions to whole-organ tissue loading. Taking these variables and complexities into account alongside the diverse test methods and equipment, it is easy to understand the broad mechanical threshold of human skin given in the literature (4.5 KPa to 30 MPa; see Supplementary File S1); Understandably, this breadth of available data makes selection of the correct modulus difficult when attempting to reproduce these values with synthetic mediums.

Despite the wealth of biomedical data on the mechanical properties of skin and skin simulants [11,12], there are no available equivalent studies that investigate the value of fibre embedded PDMS gel membranes as skin surrogates.

Skin is the largest organ of the human body, and its structure is unique to mammals. In humans, the skin is made of two behavioural membranes varying in total thickness between (0.5 mm to 4 mm), the epidermis and the dermis.

The epidermis forms the protective outer membrane while the dermis forms the softer, ‘living’ layer beneath. Both membranes work together to perform a specific function, and like most biological membranes, their function is intrinsically linked to their structure [13]. The structure of the epidermis or the *stratum corneum* is comprised of compacted, dead cells made mostly (90% to 95%) from keratin, which creates a thin, but firm membrane (<100 μm) that protects the softer, underlying dermis. A thin, ‘stiff’, viscoelastic membrane [14,15]. The dermis contains all the biologically active elements of the skin such and hair follicles, oil and sweat glands, nerve endings, blood vessels and lymph vessels; it is thicker than the epidermis (1 mm to 3 mm) more viscoelastic, softer and more extensible than the epidermis and contains large amounts of coiled collagen and elastin fibres [15].

Skin is a linear isotropic material under strains of 50% [16], while elastin is almost perfectly linear in its response to elastic deformation up to 150% and is present as thin strands in the skin. Collagen forms the leading architecture for soft tissue formation, formed from twisted helical fibrils that organise themselves into 3-dimensional tissue structure [7]. These collagen structures untangle and stretch as the skin extends, rearranging themselves back into coiled structures as the skin relaxes. There is a wealth of literature on biomechanical investigations into the physical limitations and characteristics of skin and its structures (Supplementary File S1). While the majority of studies in the literature focus on tensile testing of excised skin specimens, results vary widely, numerous contributory factors have been discussed at length in the literature, but it is agreed that the microstructure of skin, is responsible for its unique viscoelastic response to strain [11,17]. Since the skin is subject to constantly varying, two and three-dimensional strain and loading, the soft tissues are continually compressed and stretched during its movement, the stiffness must be measured alongside uni-axial and multi-axial test methods to accurately reflect the mechanical response of the skin of live subjects. It is also essential to consider the characteristic tactile response of tissue mobility and the effect of historic strain on the extensibility and elastic recoverability in cyclic tests, defined in the literature by the term ‘preconditioning’ [7]. In this paper effect of strain over time will be explored by examining hysteresis and force decay of specimens during cyclic, multi-axial tests.

Our study investigates the mechanical behaviour of fibre filled PDMS viscoelastic gel membranes, which identify with a variety of standard, repeatable mechanical tests that explore the relationship between synthetic (PDMS composites) and biological fibrous membranes (human skin). Published literature on the mechanical properties of living skin has been included for comparison in the Supplementary File S1.

Furthermore, this investigation helps identify elements of prosthetic design that remains mostly unexplored, undefined and unpublished.

We present the tactile-mechanical profile of living skin discussed in the literature and compare results of mechanical testing of synthetic surrogates produced for the purpose of this study. The elastic Young’s modulus for each surrogate is given to align this study with the contribution of other researchers, to explore the relationship between skin and synthetic skin surrogates, in terms of the mechanical characteristics of (healthy) living skin. To do this, the change in mechanical response was measured in two PDMS gel composite groups. Each composite specimen group contained 50 successfully tested specimens split into five sub-groups based on their fibre content and were prepared as single lamina sheet membranes (2 mm thickness) each containing an incremental percentage of short-strand polyester fibres (between 0% and 4%).

## 2. Results

### 2.1. Specimen Description

Throughout this study, we referred to PlatSil^®^ Gel 10 and additional components constituting a compound PDMS elastomer simply as ‘PDMS A-10’ (detailed in 4.3; Available from Mouldlife, Miro House, Western Way, Bury St Edmunds, Suffolk, UK).

In addition, we referred to the composite made from PlatSil^®^ Gel 00-30 and additional components constituting a compound PDMS elastomer, simply as ‘PDMS 00-30’ (detailed in 4.3; Available from Mouldlife, Miro House, Western Way, Bury St Edmunds, Suffolk, UK). 

The names ascribed to the two material groups throughout this study (A-10 and 00-30) refer to the initial elastomer hardness of the starting materials for each blend we prepared. It did not refer to the hardness of the material at any stage in this study, as elastomer hardness would change dependant on the additives used (detailed in 4.3). When describing the hardness of a specimen the type/hardness/test duration (in seconds) were presented. Ratios for all materials used are given in Supplementary File S2. The dimensions of fibres are given in Supplementary File S3.

### 2.2. PDMS A-10 and PDMS 00-30 Indentation Hardness

Determining soft tissue hardness using the sense of touch is a tacit craft known as palpation and has been used by experienced physicians for millennia to diagnose patient ailments. This tactility can be reliably measured and characterised by indentation shore hardness tests, using a durometer to qualitatively measure the hardness of skin (in vivo) and elastomers.

The hardness of the PDMS membranes used in this study was evaluated using an AMST standard 00 calibrated durometer. The applied force for each result was calculated using the equation given in ASTM D2240-03.

Hardness characteristics were described by average hardness (00 hardness scale) and the equivalent force was measured by the indenter spring in Newton’s.

**Figure 1:**
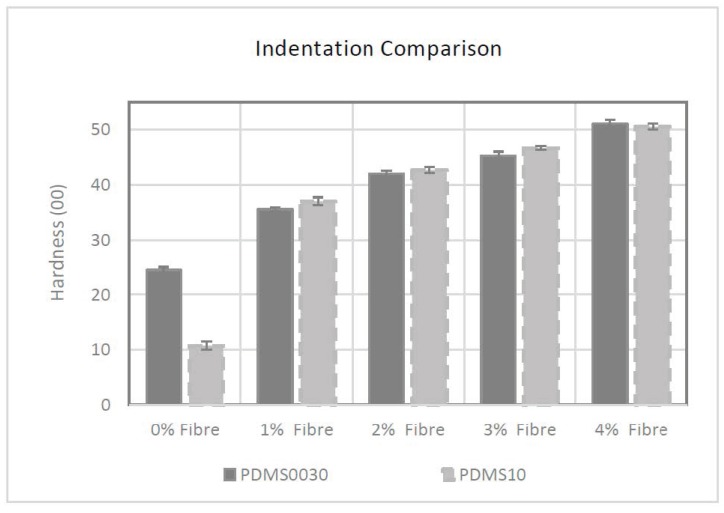
Comparison of hardness values caused by incremental fibre addition on each group of PDMS. The error bars represent the standard deviation in each group.

As can be seen in Figure 1 the control specimen group for PDMS A-10 had the lowest initial hardness of 00/10/3 after addition of the softening agent and an average force of 0.3N; however, the impact of embedded fibres was apparent when the fibre saturation was raised. The hardness value of specimens increased to 00/37/3 with an average force of 0.54 N when adding 1% fibres into the compound. Fibre saturation of 2%, 3% and 4% had elevated hardness to 00/42/3, 00/46/3 and 00/50/3 respectively with the calculated force of 0.59 N, 0.63 N and 0.66 N.

The effect of incremental fibre addition on hardness, was similar in PDMS 00-30 group too. The control specimen for this group had the lowest average hardness value of 00/24/3 and an average force of 0.42 N. By increasing the fibre saturation from 1% to 4%, specimen hardness increased from 00/35/3 to 00/51/3, and average force increased from 0.53 N to 0.67 N.

The initial hardness for the primary constituent material in the PDMS A-10 composite group was higher than PDMS 00-30 (A-10 hardness was equivalent to approximately 00/50 when placed in the same 00 scale of hardness) because of the greater ratio of softener added to A-10 composite during the liquid preparation phase (A-10 = 4:3 and 00-30 = 2:1, base silicone to softener respectively) the specimens tested by a durometer without fibres exhibited an exaggerated initial softness.

Statistical analysis of these results reveals that the presence of fibres had a proportional effect on the hardness of the PDMS membranes with a strong linear coefficient of 0.995 (y = 0.0022x − 0.0734. R² = 0.9908) from 00/37/3 to 00/51/3 for group PDMS A-10. A similarly strong relationship was observed in group PDMS 00-30 that had a positive linear coefficient of 0.993 (y = 0.002x − 0.0612. R² = 0.9875). Data and coefficient vectors for PDMS A-10 and PDMS 00-30 are given in Supplementary Files S4–S7.

### 2.3. Tensile Test Results PDMS A-10

All the specimens in both groups were tested to failure. The stress versus strain curves resulted in a comprehensive characterisation of the ultimate tensile strength (UTS) and elastic Young’s modulus, which is summarised comparatively for each conducted test in 2a and b. Elastic modulus was calculated using the rising slope of the first linear part of the deformation (0.02–0.44 MPa within 0.1–3.5 strain range) after the initial ‘toe’ of the curve created as the specimen begins to deform, but before the anisotropic phase of specimen loading. The stress versus strain curve was plotted automatically during tests using the equipment specified in 5.2.

**Figure 2:**
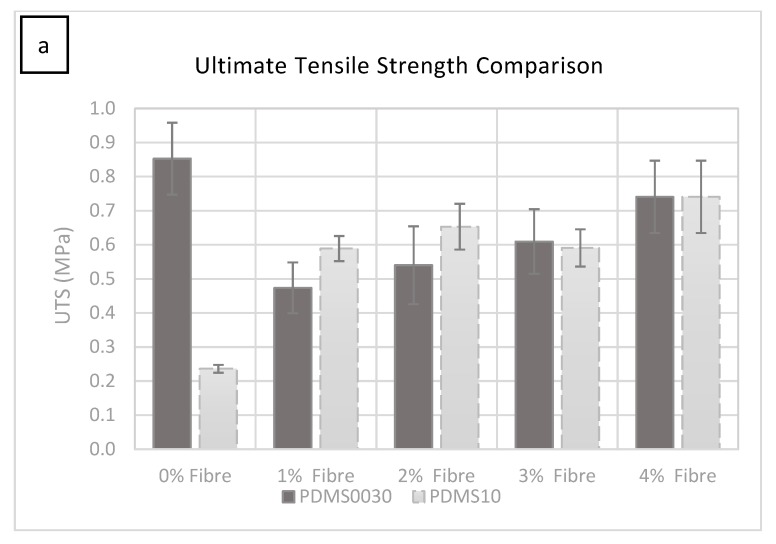

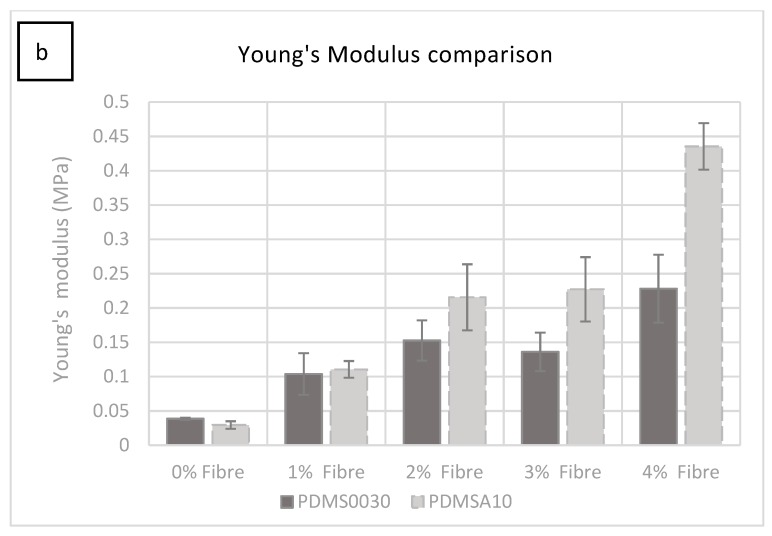
(**a**) The ultimate tensile strength comparison or force taken to rupture the specimen, given in MPa for each specimen group of PDMS A-10 and PDMS 00-30 composites with 0%–4% fibre addition. The error bars represent the standard deviation in each group. (**b**) Average Young’s modulus comparison for each specimen sample group with 0%–4% fibre addition. The error bars represent the standard deviation in each group.

As can be seen in Figure 2a, the force required to rupture the specimen (given in MPa) increased by adding more fibres. The most significant change occurred from 0% to 1% fibre saturation. A smaller but significant increase was observed in each group as the percentage of fibre saturation was increased.

The force required to rupture the specimens increased in specimens with more added fibres (1%–4% fibre addition), with a small fluctuation at 3%, which was not statistically significant. It was clear from these tests that the hardness increase was due to the increase in fibre saturation. The test specimen group with 1% fibre saturation exhibited an average value of 0.588 MPa at 1606.396% strain and had an average Young’s modulus of 0.110 MPa (Figure 2b).

The manufacturer of PlatSil^®^ products, Polytek development Corp^®^, specifies a UTS of 1.57 MPa for unmodified PlatSil^®^ gel 10. The modified blend used in this study with added softener gave a UTS of 0.236 MPa at 1310.79% strain and exhibited an average elastic Young’s modulus of 0.029 MPa, which can be seen in Figure 2b. While the ultimate tensile strength and Young’s modulus increased by adding more fibres to the compound, the strain percentage reduced from 1520.849% at 2% fibre saturation to 1459.541% at 4% fibre saturation.

Image analysis of specimens after testing to failure shows that at 0% and 1% fibre saturation no observable plastic deformation occurs, but at higher fibre concentrations permanent plastic deformation was occurring in all remaining specimens at 2%, 3% and 4% fibre saturation.

**Figure 3:**
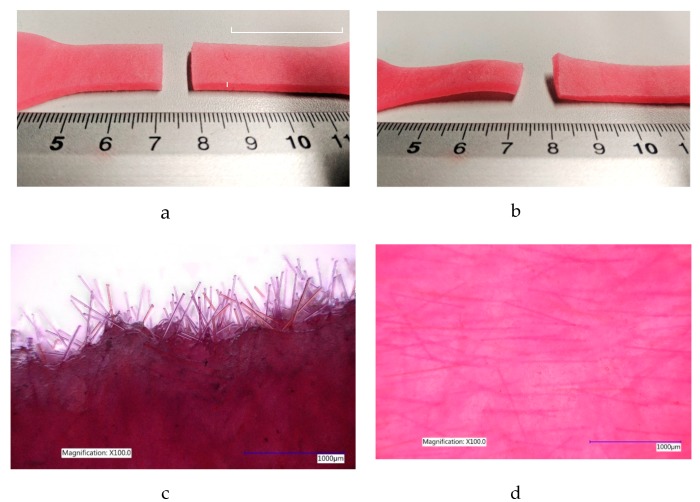
(**a**) Tensile test specimen has a clean break without permanent deformation at 0% and 1% fibre saturation. (**b**). Tensile test specimen begins to exhibit signs of permanent deformation at 2%, 3% and 4% fibre saturation. (**c**). The broken edge of specimen PDMS 00-30 containing 4% fibre saturation tensile test specimen at ×100 magnification after UTS evaluation. (**d**) The fibres appearing to orient themselves in the direction of load after specimen failure. It is important to mention that the even dispersion of fibres throughout the specimen can also be seen here.

Furthermore, the random arrangement of fibres could be seen to orient themselves in the direction of applied force within the PDMS structure when stretched to failure in specimens that exhibited permanent plastic deformation. When the specimen ruptured, fibres were seen to emerge in the direction of applied force on the distal end of the specimen shown in Figure 3c. This is consistent with the typical response of skin at high strain rates [17,18]. See Supplementary File S8, for all PDMS A-10 stress/strain graphs collected for each specimen group examined. 

Elastic modulus was calculated using the rising slope of the first linear part of the deformation, (0.04–0.23 MPa within 0.2–1.4 strain range), after the initial ‘toe’ of the curve created as the specimen begins to deform, but before the anisotropic phase of specimen loading. The stress versus strain curve was plotted automatically during tests using the equipment specified in 5.2.

The manufacturer of PDMS 00-30, Polytek Development Corp^®^, specifies a UTS of 0.81 MPa for unmodified PlatSil^®^ gel 00-30. The modified blend with added softener used in this study gave a UTS of 0.85 MPa at 1835% strain and presented an average elastic Young’s modulus of 0.037 MPa, the (Figure 2a,b).

Between 0% and 1% fibre saturation there was a significant drop in UTS before gradually increasing with the incremental addition of fibres from 1% up to 4% fibre addition.

The test specimen group with 1% fibre saturation exhibited an average UTS of 0.47 MPa at 1347% strain and showed an average Young’s modulus of 0.08 MPa.

As was seen in the previous group, the deformation of specimens at breaking point was seen to be permanent in all specimen groups with 2%, 3% and 4% fibre saturation. The initial random arrangement of fibres were, once again, seen to realign in the direction of applied force and emerged from the broken end of the specimen similarly seen in Figure 3c. See Supplementary File S9, for all PDMS 00-30 stress/strain graphs collected for each specimen group examined.

### 2.4. Multi-Axial Test Results (Force Decay and Unrecovered Deformation)

The multi-axial cyclic test (six cycles) was performed on all disc specimens up to 5 N conforming to the force threshold of 4.4 N to 8.8 N, given in the literature, replicating the exerted force on the living tissue during surgery by the surgeons’ hand tools. [19]. Force decay was calculated while the sample was held at maximum force (5 N) for 60 s at the fifth cycle.

**Figure 4:**
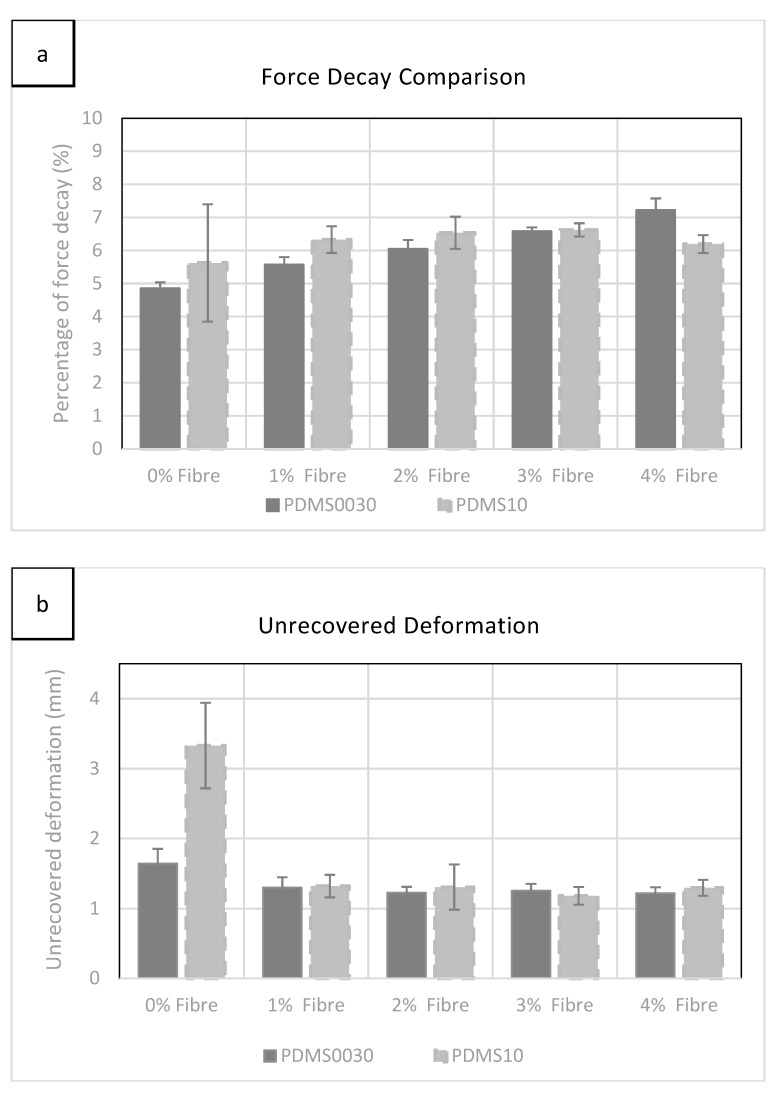
(**a**) Comparison of force decay of samples measured after 60 s on the 5th loading cycle. The error bars represent the standard deviation in each group. (**b**) Comparison of the unrecovered deformation observed in all specimens after 60 s at 5 N. The error bars represent the standard deviation in each group.

As can be seen in Figure 4a, force decay rose gradually while adding more fibres to each group with the exception of PDMS A-10 at 4%, which exhibited a small drop, which was statistically insignificant. However, a more significant change was observed in softer PDMS 00-30 specimens during the incremental addition of fibres.

Evaluation of unrecovered deformation, shown in Figure 4b, revealed little difference between groups as fibre saturation was increased. The most significant value was exhibited in specimens without added fibres. The highest level of unrecovered deformation (3.32 mm) was observed in group 00-30 with 0% fibre saturation. See Supplementary Files S10 and S11, for all force decay and unrecovered deformation graphs collected for each specimen group examined.

## 3. Discussion

This investigation aimed to characterise the mechanical behaviour of fibre filled PDMS viscoelastic gel membranes for use in surrogate skin models, [20] prosthesis [3] and other educational and research uses. The primary focus during testing was to quantify the impact of fibre saturation on two modified PDMS blends. This was achieved by using a varying concentration of fibres to change the mechanical characteristics. Mechanical profiling of human skin (Supplementary File S1) shows that the presence of fibres in the skin can provide highly variable values in shore hardness, strength, viscoelasticity and recovery. This study presents a non-clinical, non-biological skin surrogate made of loose fibres embedded in viscoelastic PDMS membranes that have some similar characteristic to those observed in living human skin. Just as fibres play a role in the fundamental behaviour of human skin, so the fibres have been shown to have a direct impact on the mechanical properties of viscoelastic PDMS membranes and can be used to with some degree of accuracy to control each of these properties.

The effect of the fibres was measured using a variety of standards (BSi, ASTM and ISO) to describe mechanical qualities observed during tests. All the materials used were sourced from commercial vendors for ease of availability and reliable repeatability.

### 3.1. Hardness

Since indentation is the only test method suitable to be done on live subjects, it enables comparative analysis of results between living skin and fibre saturated PDMS. Recorded hardness value for living human skin by durometer has been given in the literature as 00/70 to 00/86. [21]

In this study, the presence of loose fibres were shown to increase the hardness value proportional to the fibre percentage added, in both PDMS composites. It was found that for every additional percentage of added fibres, hardness increased consistently by 5 degrees of shore hardness (±1 degree of 00 shore hardness), showing that the stiffness of PDMS can be altered using the addition of fibres as opposed to adding or altering the chemical composition. This is important because varying the chemistry of the PDMS can have additional, undesirable repercussions such as unpredictable curing time and changes in mechanical properties of the composite.

Using the positive coefficient previously unknown in the literature, it is estimated that in order to reach membrane hardness of 00/70, 8.5% fibre saturation is required when using the coefficient of 0.995 for group PDMS A-10 and 8% fibre saturation is needed in group 00-30 using the coefficient of 0.993. The upper threshold of 00/86 is reached at 12% fibre saturation in group PDMS A-10 and 11% fibre saturation in group PDMS 00-30.

PDMS thinners or silicone oil can be used to aid fibre dispersion at higher fibre concentrations during preparation of the liquid elastomer. PDMS oil saturation has also been shown to create self-lubricating oily elastomers in the recreation of synthetic soft tissues, albeit at the expense of other mechanical properties, including reduction of hardness, tear-resistance and tensile strength. [22].

### 3.2. Elastic Modulus

The modulus of human skin is highly variable and is dependent on many factors such as age, sample site, orientation of Langar lines, hydration, temperature, test conditions and equipment. For this reason, considerable literature produced over the past six decades has produced very different outcomes for elastic modulus comparison (given in Supplementary File S1). 

The elastic modulus of specimens in all groups tested during this study was in good agreement with the literature and was found to be within the lower range of modulus threshold values ranging from 0.0296 MPa to 0.435 MPa (±0.033) where the literature discloses a slightly wider range of 0.014 MPa to 0.6 MPa [23,24,25]. 

UTS data for each specimen group describes the failure profile of these composites and gives the Young’s modulus. The stiffest composites containing 4% fibre saturation gave a maximum stress of 0.74 MPa for PDMS A-10 and 0.197 MPa for PDMS 00-30, which is within Young’s modulus threshold in the literature (0.0045 MPa to 221.9 MPa) [11]. Whereas this threshold value, has been more widely regarded to be within 2.5 MPa–30 MPa [24,25,26,27,28,29,30]. The lower range of modulus exhibited by the specimens tested in this study might be due to the hyper-elastic nature of soft PDMS gels used, the extensibility exaggerated further by the addition of softening agents. However, this might also be equally attributed to the lack of any internal fibrous structures like those found in biological counterparts (human skin); the loose-fibre presence affecting mainly the strength (UTS) and hardness. Similarities were found in the literature with regards to strength and hardness tests of identical PDMS composites at 2% fibre saturation where the moduli of specimens presented in this study was good agreement with the literature, but the extensibility was beyond the range given in the literature [20].

Interestingly, during the tensile tests, where specimens were tested to failure, it appeared that the fibre orientation began to align with the direction of applied force (Figure 3d). This was more apparent in specimens with higher fibre content, where the elastomeric content of the specimens was no longer high enough to deter permanent deformation. At higher concentrations of 2% fibres and above, the fibres slide out of their surrounding matrix, aligning themselves in the direction of applied force, permanently deforming the specimen due to the lack of fibre elasticity, bending the fibre outside of its initial position upon relaxation, in-turn, permanently distorting the surrounding gel matrix (Figure 3b). Similar phenomena observed in other fibre filled rubber types are discussed in more detail by Sreeja et al. 2001. [1].

The force required to permanently deform specimens was highest in groups with higher fibre saturation, but all specimens that contained fibres behaved uncharacteristically for isotropic elastomers.

**Figure 5:**
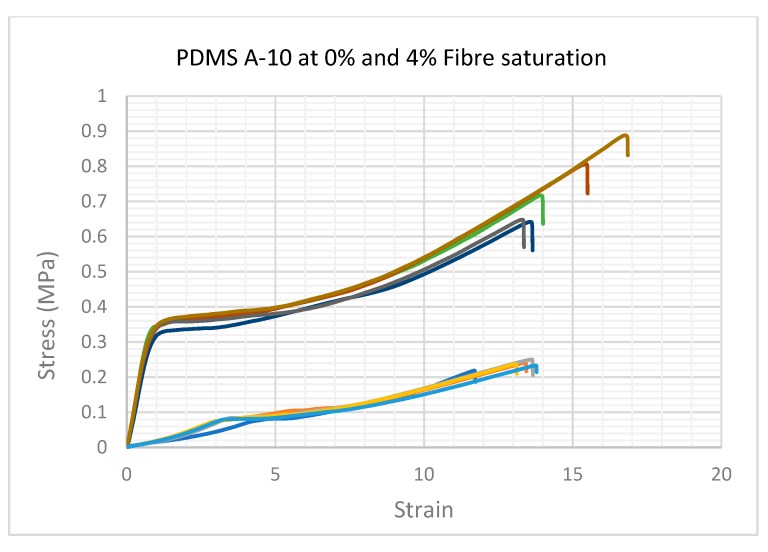
An example of the comparative stress/strain curves derived from recorded specimen data.

The change in fibre organisation could be attributed to the short linear phase of the graph with the strain value below 1, where the fibres were being rearranged and aligned in the direction of applied force, but not yet absorbing load, allowing the PDMS to exhibit a typically Hookean elastic, isotropic slope with a shorter and steeper trajectory than the following, much longer strain-hardening phase (Figure 5).

The lower group of vectors in this graph show the A-10 group with 0% fibres. The stress/strain curves are typical of Hookean elastomers, lacking fibres this group exhibited minimal signs of viscoelastic anisotropy.

The higher group of vectors show the A-10 group with 4% fibres. The exaggerated, uncharacteristic stress/strain characteristics were also observed in all specimens saturated with fibres during UTS tests. The gradient of the elastic phase increased but shortened gradually as more fibres were added.

In the next phase of the stress versus strain curve, the internal fibre matrix begins to absorb the stress, this could be seen clearly by the sharp heel formation of the curve before entering a long, anisotropic curve, forming a long and exaggerated plastic phase of permanent deformation prior to break. While this behaviour was recognised in all the specimens with more than 2% fibre saturation, the profile became more exaggerated as the fibre content was increased.

In conclusion, the presence of fibres affects the physical properties of the PDMS blend by introducing short-term strength to the elastic phase. As presented in this study, the viscoelastic anisotropic behaviour of the PDMS specimens with <1% fibre saturation had no permanent deformation as the fibre concentration was low enough not to affect the viscoelastic profile. In contrast, specimens with more than 2% fibre saturation had variable stress versus strain relationships, strongly related to fibre saturation. The tensile characteristics of living skin may be modelled more accurately by altering the extensibility and architecture of embedded fibre components to mimic the progressive alignment of fibres in the direction of applied force and the straightening of the coiled collagen and elastin fibres in the skin during extension [7].

### 3.3. Multi-Axial Tests

Three-dimensional forces exerted by surgeons’ tools during surgery is quite high (4.4–8.8 N) [19], a quantitative study of the forces exerted by the surgeons’ gloved hand when manipulating live organs is much lower. Therefore, the specimens tested during multi-axial compression were subjected to a maximum of 5 N force during cyclic tests. In PDMS A-10 blend, which included a softening agent (Smiths Theatrical Prosthetic Deadener), without fibres and has been characterised in the literature [22], it was found that the addition of fibres helped to stabilised the specimen’s physical behaviour and reduced permanent deformation at low strain percentages. However, unrecovered deformation decreased with the increment of additional fibres in both groups between 0% and 1% while the most significant drop was recorded for PDMS A-10 in this region. It was also clear that by increasing the fibre concentration above 1% does not alter the unrecoverable deformation.

On the other hand, by increasing the fibre saturation, the force decay percentage gradually increased in both groups, with a slight decrease at 4% for PDMS 10, which was not statistically significant.

In conclusion, the effect of fibres was found to have a strong relationship with the strength of the composite structure caused by the fibres as they reorient themselves in the direction of multi-axial forces, increasing recoverability after cyclic loading. In addition, by increasing the fibre saturation, the force decay was increased gradually, due to the migration of fibres within the PDMS compounds.

## 4. Method and Materials

To examine the effect of fibre saturation on elastomeric membranes, short-strand fibres were added to two base viscous liquid PDMS compounds that comprised of four specific components listed below.

### 4.1. Sample Sheet Preparation

Two different PDMS composite base liquids were blended for all mechanical testing, A-10 PDMS (PlatSil^®^ gel 10, Polytek Development Corp’, Easton, PA, USA) and 00-30 PDMS (PlatSil^®^ gel 00-30, Polytek Development Corp’, USA).

Skin simulants used in the prior art by Mahoney et al. 2018 were developed by the author (RA) as a two-layered PDMS-based composite membrane (described in this investigation as separate layers) that agreed with the hardness values of human skin and pig skin reported by [31]. Liquid ingredients and ratios for both blends are given in Supplementary File S2, but both materials similarly contained a PDMS base material (A), PDMS catalyst (B), softening agent—(Polytek Development Corp’, USA), ‘Smith’s’ theatrical prosthetic deadener (S) and cross-link retarder (Polytek Development Corp’, USA, PlatSil^®^ retarder; R). While the base elastomer ratios (50:50) remained the same for all the test specimens in both groups, for comparative analysis, the percentage of used fibres within the compound was modified with 1% increments from 1% to 4%. A control specimen group absent of embedded fibres (0%) was also included to assess the impact of fibre introduction.

### 4.2. Fibre Analysis

Commercially available, short-strand polyester fibres also included in skin simulants created in (20) were chosen due to their high absorbency, strength and flexibility in the tacit application of theatrical and maxillofacial prostheses.

**Figure 6:**
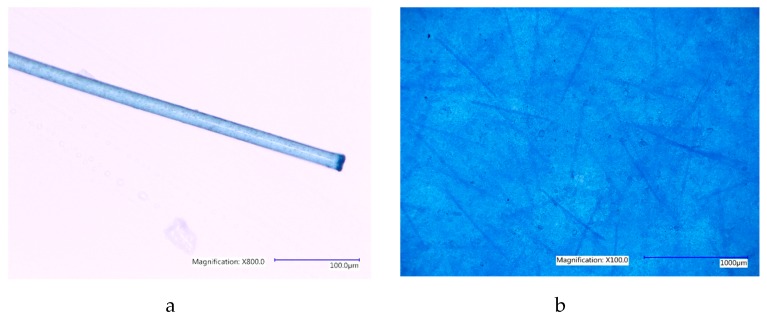
(**a**) The fibre type used in all tests shown here at ×800 magnification. The burred, cut-edge visible in the image suggests the fibres were cut with a hot blade during manufacture. (**b**). Prepared elastomeric specimen PDMS A-10 with 2% fibre saturation at ×100 magnification. Note also the even dispersion of fibres throughout the specimen without clumping of fibres.

A Keyence, VHX5000 digital microscope (Milton Keynes, UK) was used to measure the length and width of these fibres at ×800 magnification (Figure 6a). Five measurements of diameter and one measurement one of length were recorded for each of the ten randomly selected fibres. The average diameter and length for these fibres were 19.4 µm and 807.5 µm, respectively. Fibres were added to PDMS gel elastomer during the liquid phase of preparation in varying concentrations as a percentage of batch specimen weight (Figure 6b). Additional data on the fibre dimensions are available in Supplementary File S3.

### 4.3. PDMS Preparation (All the Materials Used in this Study are Commercially Available from Mouldlife, Miro House, Western Way, Bury St Edmunds, Suffolk UK) 

Both base silicone compounds (PlatSil^®^ gel 00-30 and PlatSil^®^ gel 10) contain; platinum salt <0.1%, organofunctional siloxanes 75%–100% and silica 0%–25%. 

‘Smiths’ Theatrical Prosthetic Deadener and PlatSil^®^ 71/73 Part R Retarder are commercially available materials. Their chemistry and molecular weight are protected intellectual property of Polytek Development Corporation, USA, and additional data ingredients or chemical composition is not accessible. Limited data on some physical properties of PDMS materials used in this research can be found in manufacturer’s data sheet [32]. It should also be noted that the chemistry and molecular analysis of these formulas was not the focus of this research.

**Figure 7:**
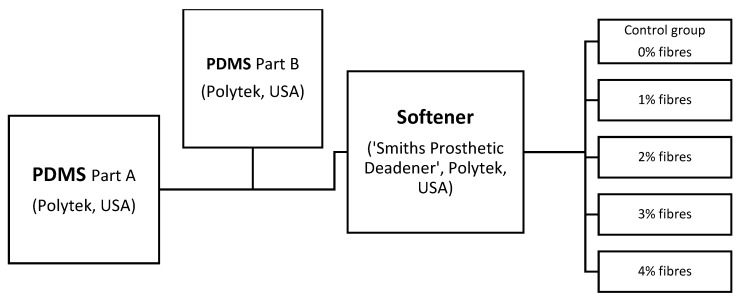
Flow diagram of the method for PDMS preparation in different fibre percentages. The same method (but different ratios) was used in all material sheet preparations. The ratios for each are given in Supplementary File S2.

Using two different colourless PMDS base components mixed according to the manufacturer’s specification, two PDMS liquid composite blends were prepared as per Figure 7.

### 4.4. Additives

Softening component (Smiths’ Theatrical Prosthetic Deadener, Mouldlife, Miro House, Western Way, Bury St Edmunds, Suffolk, UK) was added in different ratios to each silicone base material (Supplementary File S2). This softening agent is known to modify PDMS gels to behave more like skin and soft tissue by reducing typical elastic ‘snap’ while increasing viscoelastic behaviour [32].

### 4.5. Fibres

PDMS A-10 (blue) was prepared with blue flocking fibres, and PDMS 00-30 (pink) was prepared using pink flocking fibres. Choice of fibre pigmentation used in all composites blends during this study only for easy visual distinction amongst sample groups. The length and average diameter of both fibre colours used (blue and pink) were found to be identical.

### 4.6. Preparation Method

The preparation method chosen for this study replicated the preparation of identical materials in previously published literature [20] and is the most widely used method of preparation for prosthetic applications. Each composite blend was mixed by hand using a plastic beaker and wooden tongue depressor for 5 min ensuring that the fibres dispersed well and did not clump. The relatively low fibre concentration and short fibre length also helped prevent clumping and uneven dispersion. The mixture was poured into a second, clean plastic beaker and mixed again for a further 3 min to ensure thorough fibre and liquid component distribution, before being degassed at −982.052 mbar of vacuum for 5 min to remove residual air content introduced during homogenisation of the dry fibres and viscous PDMS liquids. The prepared liquid compound was poured into a levelled, 500 mm × 500 mm × 2 mm plastic gauge mould and left to cure for 48 h before being carefully removed from the tool in preparation for test specimen dissection.

All specimen groups were cut from the sample sheet using the British standard recommended for the preparation of rubber compounds (BS/ISO 23529:2016) and were powdered with talc prior to pre-test conditioning/storage for two weeks at a constant 22 °C and 50% to 60% humidity.

### 4.7. Tensile Test Specimen Preparation

ASTM D412 Type A dog bone (dumbbell) shaped stamp cut specimens for uni-axial tensile testing. This die is the largest size in either the BS/ISO 37:2011 or the ASTM D412 equivalent standard. It was selected due to the softness of the gel compound being tested in agreement with similar tests by comparing pig skin [18] and silicone rubber [33]. Smaller dies present problems with very soft specimens due to the nature of this destructive test.

### 4.8. Indentation and Multi-Axial Sample Preparation

A steel disc template of 145 mm diameter was used to cut specimens for multi-axial and indentation tests, using a surgical scalpel. A new surgical scalpel blade was used to cut each specimen group.

### 4.9. Equipment

Three mechanical tests were performed:

Indentation by durometer measured the hardness (00), uniaxial tension testing measured the elastic modulus and ultimate tensile strength (UTS) at break and multi-axial compression testing measured force decay and relaxation.

## 5. Test Methods

### 5.1. Indentation Test Method

Indentation is a common method for the characterisation of elastomers used to measure skin hardness in vivo [21,27], and is also the standard measurement method used to evaluate the hardness of elastomers.

Indentation by durometer was performed on all disc specimens to ASTM standard (D2240-03) guidelines. Although indentation by durometer was performed on all disc specimens to ASTM standard (D2240-03), it is important to mention that indentation of soft gels (<30 shore A hardness) cannot be reliably converted mathematically to Young’s modulus, so data for indentation hardness value was given in shore hardness. Due to the softness of specimens, a type 00 shore hardness calibrated durometer (‘Checkline’, Cedarhurst, NY, USA SN: 50168) with a spherical indenter tip measuring 2.5 mm in length and 2.3 mm in diameter. The durometer had a mass of 246 g, the durometer mounting assembly of the stand used during tests raised total test mass to 403g, which was exerted on each sample during tests. The stand conformed with ASTM D2240-03, type 2 stand-RX-OS-4H and controlled the rate of decent during tests. Prior to test commencement, three specimens were plied to a total thickness of 6 mm using 2 mm thick disc specimens (as specified in ASTM D 2240-03, 6.1). Each stack of specimens was measured five times, in different locations, each reading was only taken after a duration of 3 s, 6 mm apart, 12 mm from any edge and recorded directly as a shore 00 hardness value. This was then converted to a force value in Newton’s for comparative purposes.

### 5.2. Tensile Test Method

Zwick Roell Z2.5 (Ulm, Germany) tensile testing machine was used to study the physical behaviour of specimens during tensile testing. BS/ISO 37:2011 and ASTM D412 test standards were used to fix the test parameters to best suit the test specimen softness. Test standard BS ISO 5893:2002 specifies three suitable methods of measuring elongation (deflection); method A; grip separation was selected to align results with similar publications [18,33].

To prevent any slippage that can occur during the elongation of elastomeric membranes pneumatic grips with serrated jaw inserts were used [26] with grip to grip separation set to 25 mm, preload of 0.5 N and test speed at 50 mm/min.

### 5.3. Multi-Axial Test Method

The Zwick Z2.5 tensile testing machine was configured according to BS/ISO 14704-2:2007 standard for compressive multi-axial testing. Specimens were secured using method A; where a horizontally mounted, ring-clamp device whilst supported from underneath with a telescopic spacer block to mitigate the risk of the specimen weight distortion (sagging) due to unavoidable gravitational forces on the soft specimens prior to testing.

The hemispherical, Teflon probe tip had a specific diameter of 100 mm, and the inside ring clamp diameter of the test area was 120 mm.

Probe speed was fixed for all multi-axial tests at 50 mm/min. Each specimen was subjected to six cycles up to 5 N. The force-displacement curve for each specimen was collected throughout each cycle (loading and unloading) while the force decay was calculated from the fifth (unloading) curve and from the final (sixth cycle) unloading curve, permanent deformation was calculated.

## 6. Summary

While increasing fibre saturation showed a strong positive linear coefficient in all specimen groups, providing a robust predictive graph for the addition of fibres, it was found that by increasing fibre saturation in specimens, a greater effect was observed on elastic strength and yield capacity and less effect on the ultimate tensile strength.

Typical of PDMS elastomers, all specimens exhibited exaggerated plastic deformation while the uniaxial elastic limit increased as more fibres were added to the compound. It has been demonstrated that the relaxation region was greatest in specimens with higher fibre saturation, showing that increasing fibre content slows the atomic dislocation of PDMS crystalline structures prior to rupture where the reorienting fibres absorbed the stress during the loading process.

During the multi-axial investigation of specimens, reverse hysteresis phenomena was observed, which has been attributed to the loss of energy caused by the dislocation of fibres within the PDMS structure, this energy loss was reduced when the fibre saturation was increased. Viscoelastic creep was seen in all specimens during multi-axial deformation and was most significant in specimens with less added fibres. This is because the crystalline structure is reinforced by the presence of fibres.

It has been shown that embedded, loose, short-strand fibres had a measurable effect on the mechanical properties of PDMS composites and that they were able to be used to vary the mechanical properties of PDMS membranes to reflect the hardness of living human skin. However, the effect of alternate more organised fibre architectures remains unknown. Future investigations should focus on identifying the impact of interconnected/continuous, longer fibres or elastic structures that can be embedded in PDMS liquid elastomeric composites to further enhance the characteristics of PDMS membranes in the pursuit of the biomimicry of soft tissues, like living human skin, for clinical and theatrical prosthetic design applications.

## Supplementary Materials

A comprehensive catalogue of all test results are available online at https://www.mdpi.com/1996-1944/12/22/3647/s1, Supplementary File S1: Skin properties from mechanical tests, found in literature. Supplementary File S2: Ratios of the materials Supplementary. File S3 Fibre dimensions. Supplementary File S4: Indentation results for PDMS A-10. Supplementary File S5: Coefficient regression for PDMS A10 fibre influenced hardness. Supplementary File S6: Indentation results for PDMS 00-30. Supplementary File S7: Coefficient regression for PDMS 00-30 fibre influenced hardness. Supplementary File S8: Uni-axial Tensile test results for PDMS A-10. Supplementary File S9: Uni-axial Tensile test results for PDMS 00-30. Supplementary File S10: Multi-axial and Force Degradation test results for PDMS A-10. Supplementary File S11: Multi-axial and Force Degradation test results for PDMS 00-30.
