# Supplementary material for: Mechanical Behaviour of Silicone Membranes Saturated with Short Strand, Loose Polyester Fibres for Prosthetic and Rehabilitative Surrogate Skin Applications
Source: Materials (Basel). 2019 Nov 6;12(22):3647. doi: 10.3390/ma12223647 (PMC6887981; doi:10.3390/ma12223647)
Supplement: Supplementary file 1 [file materials-12-03647-s001.zip › supplementary/supplementary 1.docx]

Supplementary Materials

Mechanical Behaviour of Silicone Membranes Saturated with Short Strand, Loose Polyester Fibres for Prosthetic and Rehabilitative Surrogate Skin Applications

Richard Arm ^1,^*, Arash Shahidi ^1^ and Tilak Dias ^1^

Advanced Textiles Research Group, Flexural Composites Research Laboratory, School of Art and Design, Nottingham Trent University, Nottingham NG1 4GG, UK; arash.shahidi@ntu.ac.uk (A.S.); tilak.dias@ntu.ac.uk (T.D.)

***** Correspondence: richard.arm@ntu.ac.uk; Tel: +115-8488-6577.

Received: 4 October 2019; Accepted: 1 November 2019; Published: date

**Table 1.** Skin properties from mechanical tests on human skin, found in literature.

| Equipment/ Test | Region | Hardness/Elastic Modulus | Source Reference |
| --- | --- | --- | --- |
| Indentation Durometer Type O | Forearm | 4.5 kPa and 8 kPa Young’s Modulus | Pailler-Mattei, C.; Bec, S.; Zahouani, H. In vivo measurements of the elastic mechanical properties of human skin by indentation tests. *Med Eng.* *Phys.* 2008, *30*, 599–606. <https://www.sciencedirect.com/science/article/pii/S135045330700135X>. doi: 10.1016/j.medengphy.2007.06.011. |
| Indentation Durometer Type O  Note: At these very low hardness ranges Young’s modulus is not reliably calculated from the 0 shore hardness scale. For comparative purposes these values are converted to 00 shore hardness scale in the presented literature. | Forearm, finger, Forehead, thigh | 0–32 SH (Finger tip)  0–39 SH  (centre finger pad)  0–30 SH  (Forearm)  0–28 SH  (thigh)  0–54 SH  (forehead) | Falanga, V.; Bucalo, B. Use of a durometer to assess skin hardness. *J. Am. Acad. Dermatol.* 1993. *29*, 47–51. <https://www.sciencedirect.com/science/article/abs/pii/019096229370150R>. doi:10.1016/0190-9622(93)70150-r. |
| Indentation  Air-flow | Forearm | 6.2–14.4 KPa | Boyer, G.; Pailler-Mattei, C.; Molimard, J.; Pericoi, M.; Laquieze, S.; Zahouani, H. Non contact method for in vivo assessment of skin mechanical properties for assessing effect of ageing. *Med Eng. Phys.* 2012. *34*, 172–178. [doi:10.1016/j.medengphy.2011.07.007](https://doi.org/10.1016/j.medengphy.2011.07.007) |
| Indentation | Abdomen | 0.58–108.19 MPa | Wei, J.; Edwards, G.; Martin, D.; Huang, H.; Crichton, M,; Kendall, M. Allometric scaling of skin thickness, elasticity, viscoelasticity to mass for micro-medical device translation: from mice, rats, rabbits, pigs to humans. <https://www.ncbi.nlm.nih.gov/pubmed/29162871> doi:[10.1038/s41598-017-15830-7](https://dx.doi.org/10.1038%2Fs41598-017-15830-7) |
| Indentation  Bio-tribometer | Forearm | 8.3 > 8.5 KPa | Zahouani, H.; Pailler-Mattei, C.; Sohm, B. Varqiolu, R; Cenizo, V; Debret, R. Characterization of the mechanical properties of a dermal equivalent compared with human skin in vivo by indentation and static friction tests. Skin Research and Technology. <https://www.ncbi.nlm.nih.gov/pubmed/19152581> 15, pp 68–76. doi:10.1111/j.1600-0846.2008.00329.x |
| Ultimate tensile strength | Forehead | 3 MPa | Jacquemoud, C.; Bruyere, K.; Coret, M. Methodology to determine failure characteristics of planar soft tissues using a dynamic tensile test. *J. Biomech.* <https://www.ncbi.nlm.nih.gov/pubmed/16472812> 40, pp 468–75 doi:10.1016/j.jbiomech.2005.12.010 |
| Initial stress | Forearm, Forehead | 14 KPa (Forearm)  8 KPa  (Forehead) | Diridollou, S.; Black, D.; Lagarde, J.; Gall, Y; Berson, M; Vabre, V; Patat, F; Vaillant, L. Sex- and site-dependent variations in the thickness and mechanical properties of human skin in vivo. International *J. of Cosmet. Sci.* 2000, 6, 421–35. <https://www.ncbi.nlm.nih.gov/pubmed/18503429> |
| Tensile strength | Forehead | 3 MPa  (Forehead) | Pailler-Mattei, C.; Bec, S.; Zahouani, H. In vivo measurements of the elastic mechanical properties of human skin by indentation tests. *Med Eng. Phys.* <https://www.sciencedirect.com/science/article/pii/S135045330700135X>. 2008, *30*, 599–606. |
| Ultimate tensile strength | Thigh | 2.5 MPa (parallel to Langer lines)  600 KPa  (perpendicular to Langer lines) | Gąsior-Głogowska, M.; Komorowska, M.; Hanuza, J.; Maczka, M; Zajac, A; Ptak, M; Bedzinski, R; Kobielarz, M; Maksymowicz, K; Kuropka, P; Szotek, S. FT-Raman spectroscopic study of human skin subjected to uniaxial stress. *J. Mech. Behav. Biomed. Mater.* <https://europepmc.org/abstract/med/23290820> 2013, *18*, 240–252. doi:10.1016/j.jmbbm.2012.11.023 |
| Ultimate tensile strength | Skin | 17.9–36.5 MPa | Gallagher, A.; Ní Anniadh, A.; Bruyere, K; Ottenio, M; Xie, H; Gilchrist, M. 2012. Dynamic Tensile Properties of Human Skin. IRC-12-59 IRCOBI Conference 2012. <http://www.ircobi.org/wordpress/downloads/irc12/pdf_files/59.pdf> |
| Ultimate tensile strength | Chest | 19.4 MPa | Holzmann, H; Korting, G; Kobelt, D; Vogel, H. Studies on the mechanical properties of human skin in relation to age and sex. *Arch. Clin. Exp. Dermatol.* 1971, *239*, 355–367. PMID: 5540499 |
| Ultimate tensile strength | Skin | 4.6 MPa  20 MPa | Manschot, J.; Brakkee, A. The measurement and modelling of the mechanical properties of human skin in vivo I. *J. Biomech.* 1986, *19*, 511–515. |
