# Supplementary material for: Mechanical Behaviour of Silicone Membranes Saturated with Short Strand, Loose Polyester Fibres for Prosthetic and Rehabilitative Surrogate Skin Applications
Source: Materials (Basel). 2019 Nov 6;12(22):3647. doi: 10.3390/ma12223647 (PMC6887981; doi:10.3390/ma12223647)

Supplementary Materials

Mechanical Behaviour of Silicone Membranes Saturated with Short Strand, Loose Polyester Fibres for Prosthetic and Rehabilitative Surrogate Skin Applications

Richard Arm ^1,^*, Arash Shahidi ^1^ and Tilak Dias ^1^

Advanced Textiles Research Group, Flexural Composites Research Laboratory, School of Art and Design, Nottingham Trent University, Nottingham NG1 4GG, UK; arash.shahidi@ntu.ac.uk (A.S.); tilak.dias@ntu.ac.uk (T.D.)

***** Correspondence: richard.arm@ntu.ac.uk; Tel: +115-8488-6577.

Received: 4 October 2019; Accepted: 1 November 2019; Published: date

Multi-axial and Force Degradation test results for PDMS 00-30

The Zwick Z2.5 tensile testing machine was configured according to BS/ISO 14704-2: 2007 standard for compressive multi-axial testing. Specimens were secured using a horizontally mounted, ring-clamp device whilst supported from underneath with a telescopic spacer block to mitigate the risk of the specimen weight distortion (sagging) due to unavoidable gravitational forces on the soft specimens prior to testing. The hemispherical, Teflon probe tip had a specific diameter of 100 mm, and the inside ring clamp diameter of the test area was 120 mm. Probe speed was fixed for all multi-axial tests at 50 mm/min. Each specimen was subjected to 6 cycles up to 5 N. The force-displacement curve for each specimen was collected throughout each cycle (loading and unloading) while the force decay was calculated from the fifth (unloading) curve and from the final (sixth cycle) unloading curve, permanent deformation was calculated.


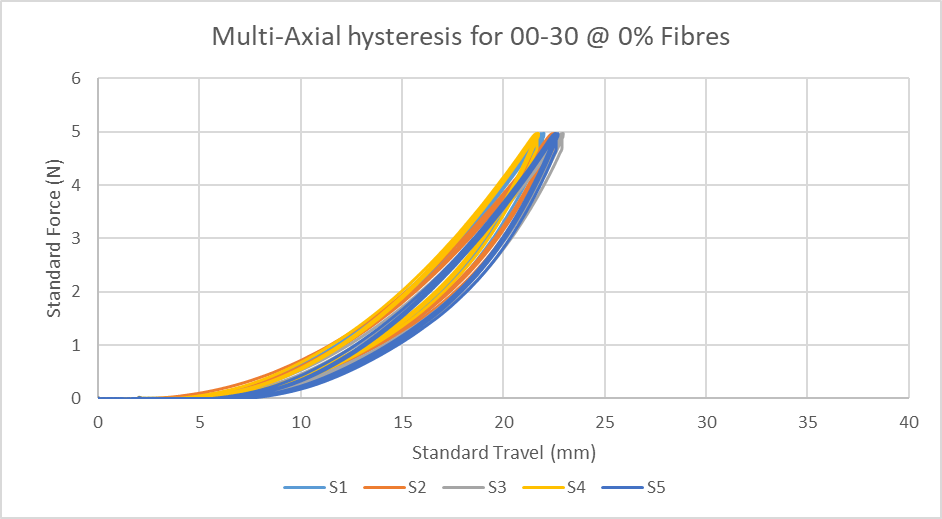


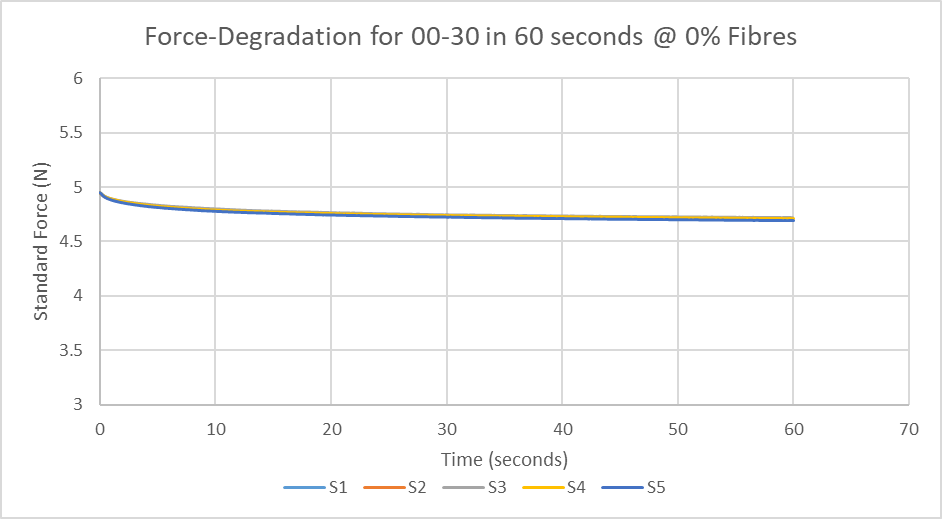


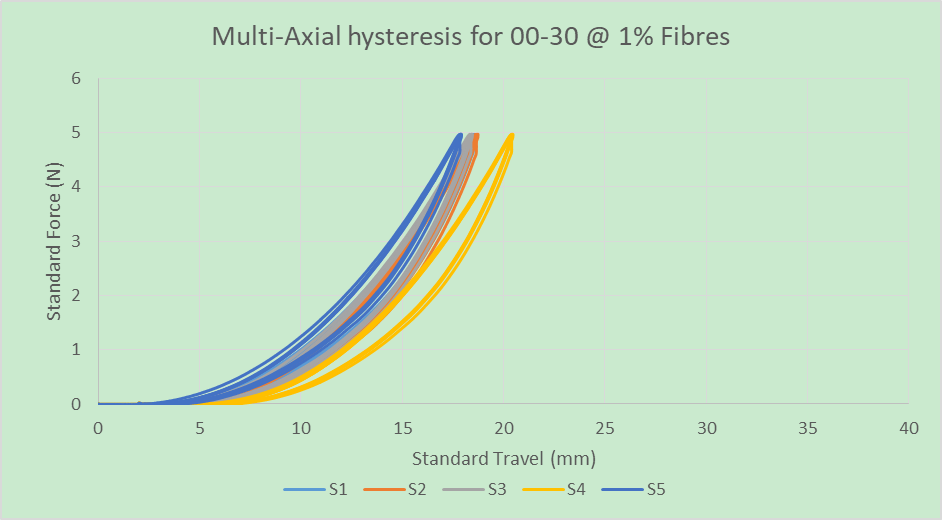


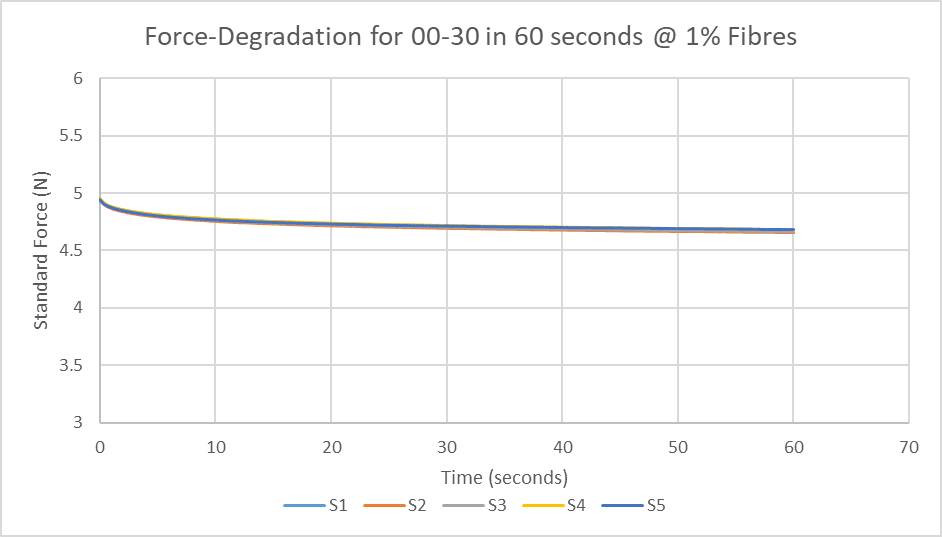


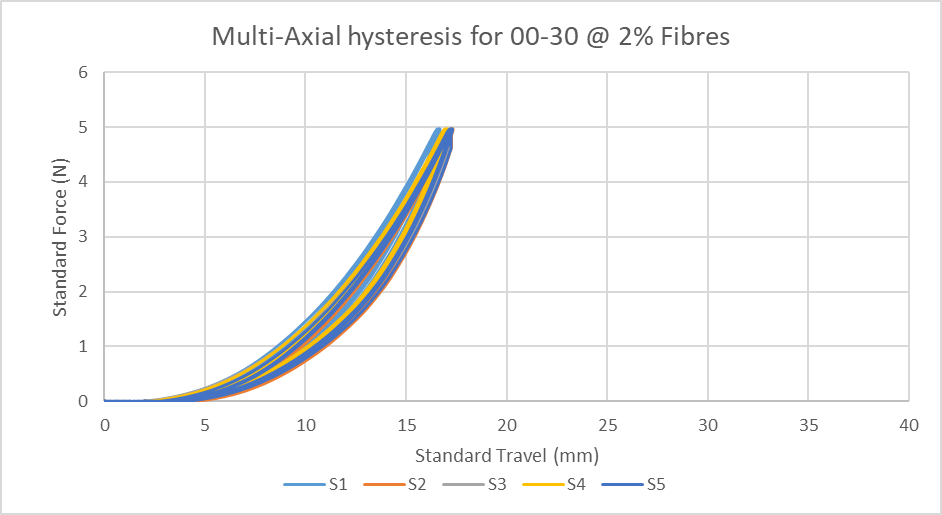


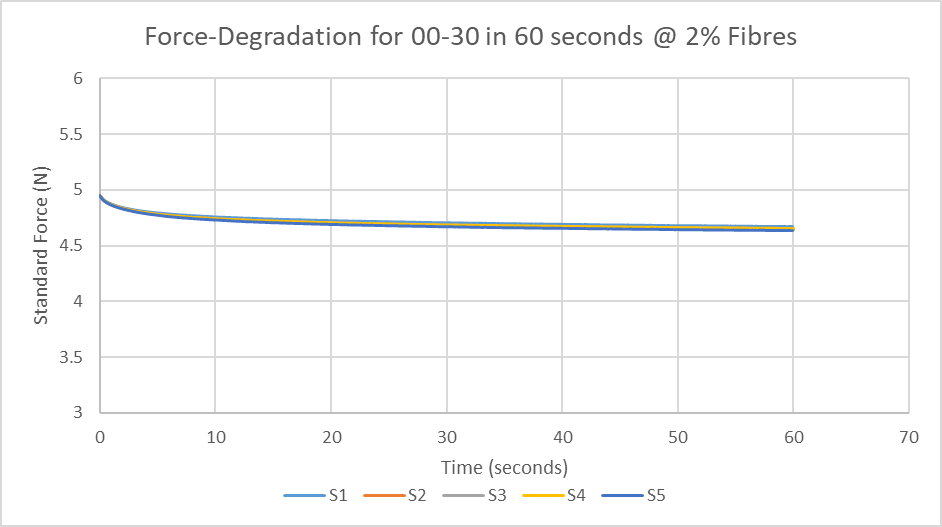


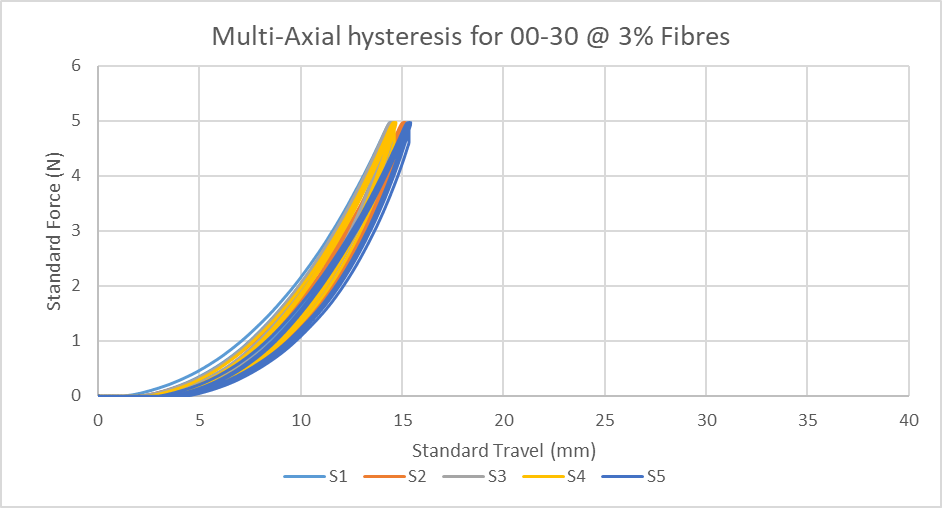


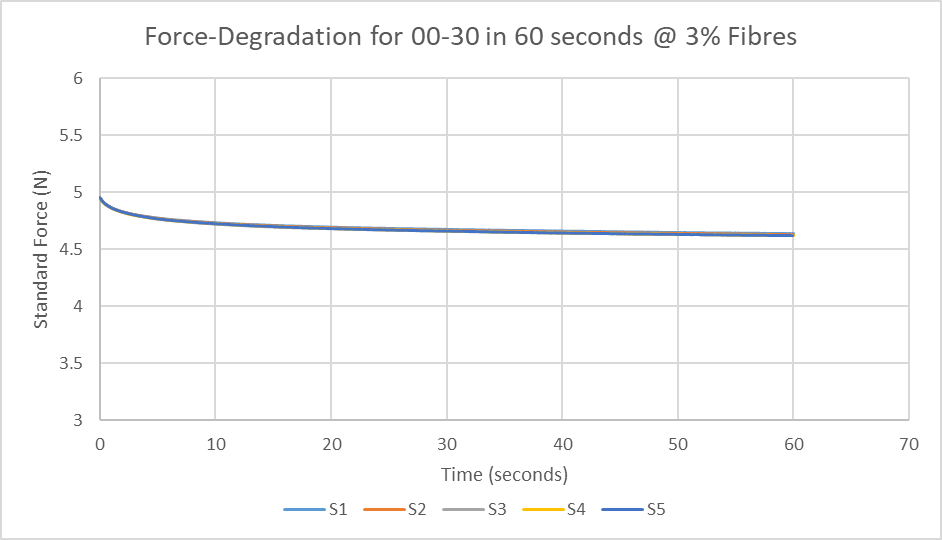


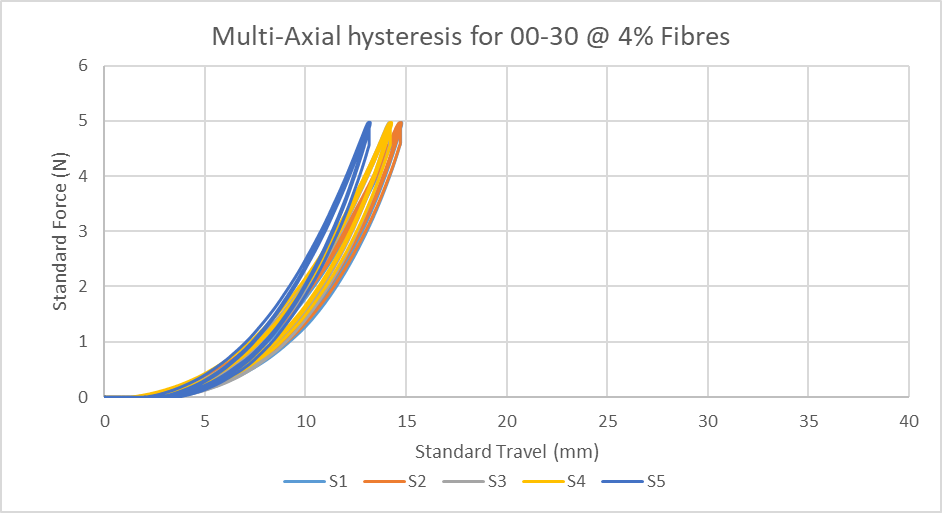


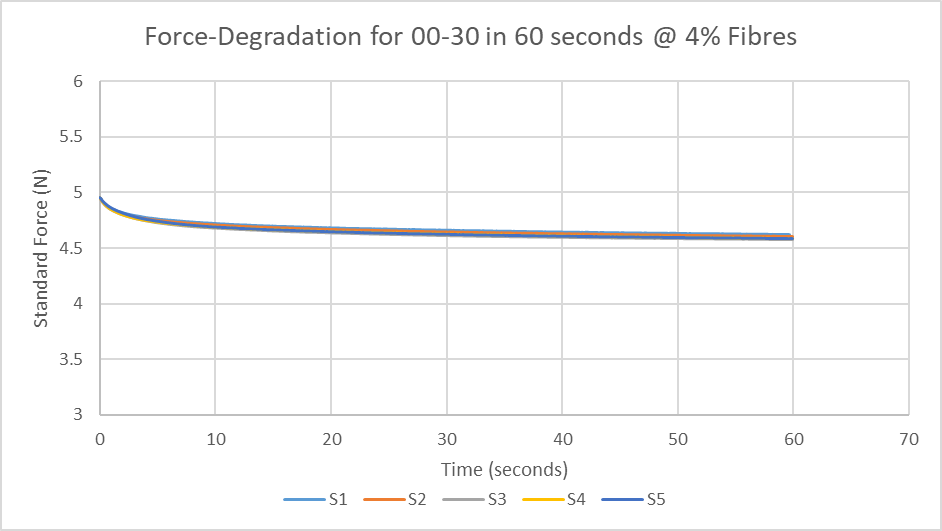

Supplement: Supplementary file 1 [file materials-12-03647-s001.zip › supplementary/supplementary 11.docx]
