# Supplementary material for: Mechanical Behaviour of Silicone Membranes Saturated with Short Strand, Loose Polyester Fibres for Prosthetic and Rehabilitative Surrogate Skin Applications
Source: Materials (Basel). 2019 Nov 6;12(22):3647. doi: 10.3390/ma12223647 (PMC6887981; doi:10.3390/ma12223647)
Supplement: Supplementary file 1 [file materials-12-03647-s001.zip › supplementary/supplementary 2.docx]

Supplementary Materials

Mechanical Behaviour of Silicone Membranes Saturated with Short Strand, Loose Polyester Fibres for Prosthetic and Rehabilitative Surrogate Skin Applications

Richard Arm ^1,^*, Arash Shahidi ^1^ and Tilak Dias ^1^

Advanced Textiles Research Group, Flexural Composites Research Laboratory, School of Art and Design, Nottingham Trent University, Nottingham NG1 4GG, UK; arash.shahidi@ntu.ac.uk (A.S.); tilak.dias@ntu.ac.uk (T.D.)

***** Correspondence: richard.arm@ntu.ac.uk; Tel: +115-8488-6577.

Received: 4 October 2019; Accepted: 1 November 2019; Published: date

Ratios of the Materials.

Each composite blend was mixed by hand using a plastic beaker and wooden tongue depressor for 5 minutes. The mixture was poured into a second, clean plastic beaker and mixed again for a further 3 minutes before being degassed at -982.052 mbar of vacuum for 5 minutes to remove residual air content introduced during homogenisation. The prepared liquid compound was poured into a levelled, 500mm x 500mm x 2mm plastic gauge mould and left to cure for 48 hours before being carefully removed from the tool in preparation for test specimen dissection.

All specimen groups were cut from the sample sheet using the British standard recommended for the preparation of rubber compounds (BS/ISO 23529:2016) and were powdered with talc prior to pre-test conditioning/storage for two weeks at a constant 22ᵒC and 50 to 60% humidity.

**Table A2.** Ratios of the materials used in this study.

| Base material: 00-30 PDMS | Base material: A-10 PDMS |
| --- | --- |
| (PlatSil® gel 00-30) | (PlatSil® gel 10) |
| Recipe | Recipe |
| 330 1 part A + 1 part B + 1 part Softener (Smiths theatrical prosthetic deadener) + Retarder (3%) | 2 parts A + 2 parts B  3 parts Softener (Smiths theatrical prosthetic deadener) + Retarder (3%) |
| 2:1 ratio (mixed PDMS: Softener) | 4:3 ratio (mixed PDMS: Softener) |
| Fibre saturation | Fibre saturation |
| 0% | 0% |
| +1% | +1% |
| +2% | +2% |
| +3% | +3% |
| +4% | +4% |
