# Supplementary material for: Mechanical Behaviour of Silicone Membranes Saturated with Short Strand, Loose Polyester Fibres for Prosthetic and Rehabilitative Surrogate Skin Applications
Source: Materials (Basel). 2019 Nov 6;12(22):3647. doi: 10.3390/ma12223647 (PMC6887981; doi:10.3390/ma12223647)
Supplement: Supplementary file 1 [file materials-12-03647-s001.zip › supplementary/supplementary 3.docx]

Supplementary Materials

Mechanical Behaviour of Silicone Membranes Saturated with Short Strand, Loose Polyester Fibres for Prosthetic and Rehabilitative Surrogate Skin Applications

Richard Arm ^1,^*, Arash Shahidi ^1^ and Tilak Dias ^1^

Advanced Textiles Research Group, Flexural Composites Research Laboratory, School of Art and Design, Nottingham Trent University, Nottingham NG1 4GG, UK; arash.shahidi@ntu.ac.uk (A.S.); tilak.dias@ntu.ac.uk (T.D.)

***** Correspondence: richard.arm@ntu.ac.uk; Tel: +115-8488-6577.

Received: 4 October 2019; Accepted: 1 November 2019; Published: date

Fibre Dimensions.

All images were recorded using a Keyence, VHX5000 digital microscope with ×1000 zoom lens.

×10 sample fibres were selected at random for fibre analysis. Each fibre was assigned a group designation number. Each fibre length was recorded and is given at the top of each group.

×5 diameter widths were recorded at random increments along each fibre length and an average fibre width is given at the bottom of each group. Measurements were recorded visually and included a scale and magnification legend for reference on each image.

| **Group 1- Length876.3 µm** | | | | **Group 2-Length 840.8 µm** | | | |
| --- | --- | --- | --- | --- | --- | --- | --- |
| **No.** | **Measure** | **Result** | **Unit** | **No.** | **Measure** | **Result** | **Unit** |
| 1 | 2 Points | 18 | µm | 1 | 2 Points | 19.5 | µm |
| 2 | 2 Points | 17 | µm | 2 | 2 Points | 19.2 | µm |
| 3 | 2 Points | 17.9 | µm | 3 | 2 Points | 18.9 | µm |
| 4 | 2 Points | 18.1 | µm | 4 | 2 Points | 19.3 | µm |
| 5 | 2 Points | 18.4 | µm | 5 | 2 Points | 19.4 | µm |
| **Average 17.88 µm** | | | | **Average 19.26 µm** | | | |
|  | | | | | | | |
| **Group 3-Length 845.6 µm** | | | | **Group 4-Length 691.2 µm** | | | |
| **No.** | **Measure** | **Result** | **Unit** | **No.** | **Measure** | **Result** | **Unit** |
| 1 | 2 Points | 21.1 | µm | 1 | 2 Points | 18.8 | µm |
| 2 | 2 Points | 20.2 | µm | 2 | 2 Points | 18.4 | µm |
| 3 | 2 Points | 19.7 | µm | 3 | 2 Points | 18.2 | µm |
| 4 | 2 Points | 20.2 | µm | 4 | 2 Points | 18.2 | µm |
| 5 | 2 Points | 20.2 | µm | 5 | 2 Points | 18.4 | µm |
| **Average 20.28 µm** | | | | **Average 18.4 µm** | | | |
|  | | | | | | | |
| **Group 5-Length 836.1 µm** | | | | **Group 6-Length 804.4 µm** | | | |
| **No.** | **Measure** | **Result** | **Unit** | **No.** | **Measure** | **Result** | **Unit** |
| 1 | 2 Points | 19.5 | µm | 1 | 2 Points | 20.9 | µm |
| 2 | 2 Points | 19.3 | µm | 2 | 2 Points | 21.1 | µm |
| 3 | 2 Points | 19 | µm | 3 | 2 Points | 20.5 | µm |
| 4 | 2 Points | 19.2 | µm | 4 | 2 Points | 21.3 | µm |
| 5 | 2 Points | 19 | µm | 5 | 2 Points | 21.3 | µm |
| **Average 19.2 µm** | | | | **Average 21.02 µm** | | | |
|  | | | | | | | |
| **Group 7-Length 792.7 µm** | | | | **Group 8-Length 940.4 µm** | | | |
| **No.** | **Measure** | **Result** | **Unit** | **No.** | **Measure** | **Result** | **Unit** |
| 1 | 2 Points | 19.9 | µm | 1 | 2 Points | 18.8 | µm |
| 2 | 2 Points | 19.1 | µm | 2 | 2 Points | 18 | µm |
| 3 | 2 Points | 19.2 | µm | 3 | 2 Points | 19 | µm |
| 4 | 2 Points | 19.9 | µm | 4 | 2 Points | 19.4 | µm |
| 5 | 2 Points | 20.3 | µm | 5 | 2 Points | 18.6 | µm |
| **Average 19.68 µm** | | | | **Average 18.76 µm** | | | |
|  | | | | | | | |
| **Group 9-Length 733.4 µm** | | | | **Group 10-Length 714.2 µm** | | | |
| **No.** | **Measure** | **Result** | **Unit** | **No.** | **Measure** | **Result** | **Unit** |
| 1 | 2 Points | 18.5 | µm | 1 | 2 Points | 21.7 µm | µm |
| 2 | 2 Points | 18.8 | µm | 2 | 2 Points | 21.1 µm | µm |
| 3 | 2 Points | 18.7 | µm | 3 | 2 Points | 20.9 µm | µm |
| 4 | 2 Points | 18.6 | µm | 4 | 2 Points | 21.7 µm | µm |
| 5 | 2 Points | 18.1 | µm | 5 | 2 Points | 21.8 µm | µm |
| **Average 18.51 µm** | | | | **Average 21.44 µm** | | | |
| **Total average diameter = 19.44 µm Total average length = 807.5 µm** | | | | | | | |
