# Supplementary material for: Mechanical Behaviour of Silicone Membranes Saturated with Short Strand, Loose Polyester Fibres for Prosthetic and Rehabilitative Surrogate Skin Applications
Source: Materials (Basel). 2019 Nov 6;12(22):3647. doi: 10.3390/ma12223647 (PMC6887981; doi:10.3390/ma12223647)
Supplement: Supplementary file 1 [file materials-12-03647-s001.zip › supplementary/supplementary 4.docx]

Supplementary Materials

Mechanical Behaviour of Silicone Membranes Saturated with Short Strand, Loose Polyester Fibres for Prosthetic and Rehabilitative Surrogate Skin Applications

Richard Arm ^1,^*, Arash Shahidi ^1^ and Tilak Dias ^1^

Advanced Textiles Research Group, Flexural Composites Research Laboratory, School of Art and Design, Nottingham Trent University, Nottingham NG1 4GG, UK; arash.shahidi@ntu.ac.uk (A.S.); tilak.dias@ntu.ac.uk (T.D.)

***** Correspondence: richard.arm@ntu.ac.uk; Tel: +115-8488-6577.

Received: 4 October 2019; Accepted: 1 November 2019; Published: date

Indentation Results for PDMS A-10.

Indentation is a common method for the characterisation of elastomers used to measure skin hardness in vivo [21,27], and is also the standard measurement method used to evaluate the hardness of elastomers. Indentation by durometer was performed on all disc specimens to ASTM standard (D2240-03) guidelines. Due to the softness of specimens, a type 00 Shore hardness (H 00) calibrated durometer (‘Checkline', USA SN: 50168) with a spherical indenter tip measuring 2.5 mm in length and 2.3 mm in diameter and the device and mounting assembly arm had a total weight of 400 g. Each reading was taken using 6mm, triple-plied, 3 × 2mm specimens cut from the same sheet material. Each plied specimen was measured five times in different locations taken 6mm apart (12 mm from any edge) and recorded directly as a shore 00 hardness value before being converted to a force value in Newton’s.

| **Test equipment** | **HOO Harness reading from Durometer (00)** |
| --- | --- |
| **Conversion factor** | **F(N) = 0.203+0.00908*H00** |
| **Formulation** | **PDMS A-10 + Softener + Fibres** |

| **Key** | **Control Group (0% Fibre addition)** | | | | | | | | | | **Average Median** | |
| --- | --- | --- | --- | --- | --- | --- | --- | --- | --- | --- | --- | --- |
|  | **Specimen 1** | | **Specimen 2** | | **Specimen 3** | | **Specimen 4** | | **Specimen 5** | | H 00 | Force (N) |
|  | **H00** | **F(N)** | **H00** | **F(N)** | **H00** | **F(N)** | **H00** | **F(N)** | **H00** | **F(N)** |  |  |
| 1 | 11 | 0.31 | 11 | 0.30 | 11 | 0.30 | 10.5 | 0.30 | 11 | 0.30 |  |  |
| 2 | 9 | 0.28 | 11 | 0.30 | 12 | 0.31 | 9 | 0.28 | 11 | 0.30 |  |  |
| 3 | 12 | 0.31 | 12 | 0.31 | 11 | 0.30 | 9 | 0.29 | 10 | 0.29 |  |  |
| 4 | 11 | 0.30 | 11 | 0.31 | 10 | 0.30 | 10 | 0.29 | 11 | 0.30 |  |  |
| 5 | 11 | 0.30 | 12 | 0.31 | 10 | 0.30 | 9 | 0.28 | 11 | 0.30 |  |  |
| Average | 10 | 0.30 | 11 | 0.31 | 11 | 0.30 | 9 | 0.29 | 10 | 0.30 | 10 | 0.30 |
| Median | 11 | 0.30 | 11 | 0.31 | 11 | 0.30 | 9 | 0.29 | 11 | 0.30 | 10 | 0.30 |

| **Key** | **Specimen Group 1 (1% Fibre addition)** | | | | | | | | | | **Average Median** | |
| --- | --- | --- | --- | --- | --- | --- | --- | --- | --- | --- | --- | --- |
|  | **Specimen 1** | | **Specimen 2** | | **Specimen 3** | | **Specimen 4** | | **Specimen 5** | | H 00 | Force (N) |
|  | **H00** | **F(N)** | **H00** | **F(N)** | **H00** | **F(N)** | **H00** | **F(N)** | **H00** | **F(N)** |  |  |
| 1 | 37 | 0.54 | 38 | 0.55 | 36 | 0.53 | 36 | 0.53 | 37 | 0.54 |  |  |
| 2 | 37 | 0.54 | 38 | 0.55 | 36 | 0.53 | 36 | 0.53 | 37 | 0.54 |  |  |
| 3 | 37 | 0.54 | 38 | 0.55 | 37 | 0.54 | 36 | 0.53 | 37 | 0.54 |  |  |
| 4 | 36 | 0.53 | 38 | 0.55 | 37 | 0.54 | 36 | 0.53 | 37 | 0.54 |  |  |
| 5 | 36 | 0.53 | 38 | 0.55 | 37 | 0.54 | 36 | 0.53 | 37 | 0.54 |  |  |
| Average | 36 | 0.54 | 38 | 0.55 | 36 | 0.54 | 36 | 0.53 | 37 | 0.54 | 37 | 0.54 |
| Median | 37 | 0.54 | 38 | 0.55 | 37 | 0.54 | 36 | 0.53 | 37 | 0.54 | 37 | 0.54 |

| **Key** | **Specimen Group 2 (2% Fibre addition)** | | | | | | | | | | **Average Median** | |
| --- | --- | --- | --- | --- | --- | --- | --- | --- | --- | --- | --- | --- |
|  | **Specimen 1** | | **Specimen 2** | | **Specimen 3** | | **Specimen 4** | | **Specimen 5** | | H 00 | Force (N) |
|  | **H00** | **F(N)** | **H00** | **F(N)** | **H00** | **F(N)** | **H00** | **F(N)** | **H00** | **F(N)** |  |  |
| 1 | 41 | 0.58 | 44 | 0.60 | 41 | 0.58 | 41 | 0.58 | 41 | 0.58 |  |  |
| 2 | 42 | 0.59 | 44 | 0.60 | 42 | 0.58 | 42 | 0.58 | 42 | 0.58 |  |  |
| 3 | 43 | 0.59 | 42 | 0.58 | 43 | 0.59 | 42 | 0.58 | 42 | 0.59 |  |  |
| 4 | 43 | 0.59 | 44 | 0.60 | 43 | 0.60 | 42 | 0.59 | 43 | 0.59 |  |  |
| 5 | 43 | 0.60 | 44 | 0.60 | 44 | 0.60 | 43 | 0.59 | 43 | 0.59 |  |  |
| Average | 42 | 0.59 | 43 | 0.60 | 42 | 0.59 | 42 | 0.59 | 42 | 0.59 | 42 | 0.59 |
| Median | 43 | 0.59 | 44 | 0.60 | 43 | 0.59 | 42 | 0.58 | 42 | 0.59 | 42 | 0.59 |

| **Key** | **Specimen Group 3 (3% Fibre addition)** | | | | | | | | | | **Average Median** | |
| --- | --- | --- | --- | --- | --- | --- | --- | --- | --- | --- | --- | --- |
|  | **Specimen 1** | | **Specimen 2** | | **Specimen 3** | | **Specimen 4** | | **Specimen 5** | | H 00 | Force (N) |
|  | **H00** | **F(N)** | **H00** | **F(N)** | **H00** | **F(N)** | **H00** | **F(N)** | **H00** | **F(N)** |  |  |
| 1 | 44 | 0.61 | 45 | 0.61 | 45 | 0.61 | 46 | 0.62 | 46 | 0.62 |  |  |
| 2 | 45 | 0.62 | 45 | 0.62 | 47 | 0.63 | 47 | 0.63 | 47 | 0.63 |  |  |
| 3 | 46 | 0.62 | 47 | 0.63 | 47 | 0.63 | 46 | 0.62 | 47 | 0.63 |  |  |
| 4 | 47 | 0.63 | 47 | 0.63 | 48 | 0.64 | 48 | 0.64 | 46 | 0.62 |  |  |
| 5 | 48 | 0.64 | 48 | 0.64 | 47 | 0.63 | 48 | 0.64 | 46 | 0.62 |  |  |
| Average | 46 | 0.62 | 46 | 0.63 | 47 | 0.63 | 47 | 0.63 | 46 | 0.63 | 46 | 0.63 |
| Median | 46 | 0.62 | 47 | 0.63 | 47. | 0.63 | 47 | 0.63 | 46 | 0.62 | 46 | 0.63 |

| **Key** | **Specimen Group 4 (4% Fibre addition)** | | | | | | | | | | **Average Median** | |
| --- | --- | --- | --- | --- | --- | --- | --- | --- | --- | --- | --- | --- |
|  | **Specimen 1** | | **Specimen 2** | | **Specimen 3** | | **Specimen 4** | | **Specimen 5** | | H 00 | Force (N) |
|  | **H00** | **F(N)** | **H00** | **F(N)** | **H00** | **F(N)** | **H00** | **F(N)** | **H00** | **F(N)** |  |  |
| 1 | 50 | 0.66 | 49 | 0.65 | 49 | 0.65 | 48 | 0.64 | 50 | 0.66 |  |  |
| 2 | 52 | 0.68 | 50 | 0.66 | 50 | 0.66 | 51 | 0.67 | 51 | 0.67 |  |  |
| 3 | 50 | 0.66 | 50 | 0.66 | 51 | 0.67 | 50 | 0.66 | 51 | 0.67 |  |  |
| 4 | 52 | 0.68 | 51 | 0.67 | 52 | 0.68 | 49 | 0.65 | 52 | 0.68 |  |  |
| 5 | 50 | 0.66 | 50 | 0.66 | 50 | 0.66 | 52 | 0.68 | 52 | 0.68 |  |  |
| Average | 50 | 0.67 | 50 | 0.66 | 50 | 0.66 | 50 | 0.66 | 51 | 0.67 | 50 | 0.66 |
| Median | 50 | 0.66 | 50 | 0.66 | 50 | 0.66 | 50 | 0.66 | 51 | 0.67 | 50 | 0.66 |
