# Supplementary material for: Mechanical Behaviour of Silicone Membranes Saturated with Short Strand, Loose Polyester Fibres for Prosthetic and Rehabilitative Surrogate Skin Applications
Source: Materials (Basel). 2019 Nov 6;12(22):3647. doi: 10.3390/ma12223647 (PMC6887981; doi:10.3390/ma12223647)
Supplement: Supplementary file 1 [file materials-12-03647-s001.zip › supplementary/supplementary 6.docx]

Supplementary Materials

Mechanical Behaviour of Silicone Membranes Saturated with Short Strand, Loose Polyester Fibres for Prosthetic and Rehabilitative Surrogate Skin Applications

Richard Arm ^1,^*, Arash Shahidi ^1^ and Tilak Dias ^1^

Advanced Textiles Research Group, Flexural Composites Research Laboratory, School of Art and Design, Nottingham Trent University, Nottingham NG1 4GG, UK; arash.shahidi@ntu.ac.uk (A.S.); tilak.dias@ntu.ac.uk (T.D.)

***** Correspondence: richard.arm@ntu.ac.uk; Tel: +115-8488-6577.

Received: 4 October 2019; Accepted: 1 November 2019; Published: date

Indentation Results for PDMS 00-30.

| **Test equipment** | | | | | | | **HOO Harness reading from Durometer (00)** | | | | | | | | |
| --- | --- | --- | --- | --- | --- | --- | --- | --- | --- | --- | --- | --- | --- | --- | --- |
| **Conversion factor** | | | | | | | **F(N) = 0.203 + 0.00908 × H00** | | | | | | | | |
| **Formulation** | | | | | | | **PDMS 00-30 + Softener + Fibres** | | | | | | | | |
| **Key** | **Control Group (0% Fibre addition)** | | | | | | | | | | | | | **Average Median** | |
|  | **Specimen 1** | | | **Specimen 2** | | | | **Specimen 3** | | **Specimen 4** | | **Specimen 5** | | H 00 | Force (N) |
|  | **H 00** | **F(N)** | | **H 00** | | **F(N)** | | **H 00** | **F(N)** | **H 00** | **F(N)** | **H 00** | **F(N)** |  |  |
| 1 | 23 | 0.41 | | 24 | | 0.43 | | 26 | 0.44 | 25 | 0.43 | 24 | 0.42 |  |  |
| 2 | 23 | 0.42 | | 24 | | 0.42 | | 25 | 0.43 | 23 | 0.42 | 24 | 0.43 |  |  |
| 3 | 24 | 0.42 | | 24 | | 0.43 | | 24 | 0.43 | 23 | 0.41 | 25 | 0.43 |  |  |
| 4 | 24 | 0.42 | | 24 | | 0.42 | | 26 | 0.44 | 26 | 0.44 | 25 | 0.43 |  |  |
| 5 | 24 | 0.42 | | 24 | | 0.43 | | 25 | 0.43 | 25 | 0.43 | 23 | 0.42 |  |  |
| Average | 23 | 0.42 | | 24 | | 0.42 | | 25 | 0.43 | 24 | 0.43 | 24 | 0.42 | 24 | 0.42 |
| Median | 23 | 0.42 | | 24 | | 0.42 | | 25 | 0.43 | 24 | 0.42 | 24 | 0.42 | 24 | 0.42 |
|  |  |  | | |  |  | |  |  |  |  |  |  |  |  |
| **Key** |  | **Specimen Group 1 (1% Fibre addition)** | | | | | | | | | | | | **Average Median** | |
|  | **Specimen 1** | | | **Specimen 2** | | | | **Specimen 3** | | **Specimen 4** | | **Specimen 5** | | H 00 | Force (N) |
|  | **H 00** | **F(N)** | | **H 00** | | **F(N)** | | **H 00** | **F(N)** | **H 00** | **F(N)** | **H 00** | **F(N)** |  |  |
| 1 | 35 | 0.52 | | 35 | | 0.52 | | 35 | 0.53 | 35 | 0.53 | 36 | 0.53 |  |  |
| 2 | 36 | 0.53 | | 35 | | 0.53 | | 36 | 0.53 | 36 | 0.53 | 36 | 0.53 |  |  |
| 3 | 35 | 0.52 | | 35 | | 0.52 | | 35 | 0.52 | 34 | 0.52 | 36 | 0.53 |  |  |
| 4 | 36 | 0.53 | | 35 | | 0.53 | | 36 | 0.53 | 36 | 0.53 | 36 | 0.53 |  |  |
| 5 | 36 | 0.53 | | 35 | | 0.53 | | 34 | 0.51 | 36 | 0.53 | 36 | 0.53 |  |  |
| Average | 35 | 0.53 | | 35 | | 0.52 | | 35 | 0.52 | 35 | 0.53 | 36 | 0.53 | 35 | 0.52 |
| Median | 35 | 0.53 | | 35 | | 0.52 | | 35 | 0.53 | 35 | 0.53 | 36 | 0.53 | 36 | 0.52 |
|  |  |  |  | |  |  | |  |  |  |  |  |  |  |  |
| **Key** |  | **Specimen Group 2 (2% Fibre addition)** | | | | | | | | | | | | **Average Median** | |
|  | **Specimen 1** | | | **Specimen 2** | | | | **Specimen 3** | | **Specimen 4** | | **Specimen 5** | | H 00 | Force (N) |
|  | **H 00** | **F(N)** | | **H 00** | | **F(N)** | | **H 00** | **F(N)** | **H 00** | **F(N)** | **H 00** | **F(N)** |  |  |
| 1 | 41 | 0.58 | | 41 | | 0.58 | | 42 | 0.58 | 43 | 0.59 | 42 | 0.58 |  |  |
| 2 | 42 | 0.58 | | 41 | | 0.58 | | 43 | 0.59 | 41 | 0.58 | 41 | 0.58 |  |  |
| 3 | 42 | 0.58 | | 42 | | 0.58 | | 43 | 0.60 | 42 | 0.59 | 42 | 0.58 |  |  |
| 4 | 42 | 0.58 | | 42 | | 0.58 | | 42 | 0.59 | 43 | 0.59 | 42 | 0.58 |  |  |
| 5 | 40 | 0.57 | | 41 | | 0.58 | | 42 | 0.59 | 42 | 0.59 | 43 | 0.59 |  |  |
| Average | 41 | 0.58 | | 41 | | 0.58 | | 42 | 0.59 | 42 | 0.59 | 41 | 0.58 | 41 | 0.58 |
| Median | 42 | 0.58 | | 41 | | 0.58 | | 42 | 0.59 | 42 | 0.59 | 42 | 0.58 | 42 | 0.58 |
|  |  |  |  | |  |  | |  |  |  |  |  |  |  |  |
| **Key** |  | **Specimen Group 3 (3% Fibre addition)** | | | | | | | | | | | | **Average Median** | |
|  | **Specimen 1** | | | **Specimen 2** | | | | **Specimen 3** | | **Specimen 4** | | **Specimen 5** | | H 00 | Force (N) |
|  | **H 00** | **F(N)** | | | **H 00** | **F(N)** | | **H 00** | **F(N)** | **H 00** | **F(N)** | **H 00** | **F(N)** |  |  |
| 1 | 45 | 0.61 | | | 46 | 0.62 | | 45 | 0.61 | 46 | 0.62 | 45 | 0.61 |  |  |
| 2 | 45 | 0.61 | | | 44 | 0.60 | | 45 | 0.61 | 45 | 0.61 | 46 | 0.62 |  |  |
| 3 | 43 | 0.60 | | | 46 | 0.62 | | 45 | 0.61 | 45 | 0.61 | 47 | 0.63 |  |  |
| 4 | 44 | 0.60 | | | 47 | 0.63 | | 45 | 0.61 | 46 | 0.62 | 46 | 0.62 |  |  |
| 5 | 44 | 0.60 | | | 46 | 0.62 | | 44 | 0.60 | 47 | 0.63 | 45 | 0.61 |  |  |
| Average | 44 | 0.61 | | | 45 | 0.62 | | 44 | 0.61 | 45 | 0.62 | 45 | 0.62 | 45 | 0.61 |
| Median | 44 | 0.61 | | | 46 | 0.62 | | 45 | 0.61 | 45 | 0.62 | 46 | 0.62 | 45 | 0.61 |
|  |  |  |  | |  |  | |  |  |  |  |  |  |  |  |
| **Key** |  | **Specimen Group 4 (4% Fibre addition)** | | | | | | | | | | | | **Average Median** | |
|  | **Specimen 1** | | | **Specimen 2** | | | | **Specimen 3** | | **Specimen 4** | | **Specimen 5** | | H 00 | Force (N) |
|  | **H 00** | **F(N)** | | | **H 00** | **F(N)** | | **H 00** | **F(N)** | **H 00** | **F(N)** | **H 00** | **F(N)** |  |  |
| 1 | 52 | 0.68 | | | 49 | 0.65 | | 52 | 0.68 | 53 | 0.68 | 49 | 0.65 |  |  |
| 2 | 52 | 0.68 | | | 51 | 0.67 | | 53 | 0.68 | 51 | 0.67 | 52 | 0.68 |  |  |
| 3 | 52 | 0.68 | | | 50 | 0.66 | | 53 | 0.69 | 51 | 0.67 | 52 | 0.68 |  |  |
| 4 | 50 | 0.66 | | | 49 | 0.65 | | 50 | 0.66 | 51 | 0.67 | 51 | 0.67 |  |  |
| 5 | 51 | 0.67 | | | 51 | 0.67 | | 49 | 0.65 | 51 | 0.67 | 50 | 0.66 |  |  |
| Average | 51 | 0.67 | | | 50 | 0.66 | | 51 | 0.67 | 51 | 0.67 | 50 | 0.66 | 51 | 0.66 |
| Median | 52 | 0.68 | | | 49 | 0.65 | | 52 | 0.68 | 51 | 0.67 | 51 | 0.67 | 50 | 0.66 |
