# Supplementary material for: Mechanical Behaviour of Silicone Membranes Saturated with Short Strand, Loose Polyester Fibres for Prosthetic and Rehabilitative Surrogate Skin Applications
Source: Materials (Basel). 2019 Nov 6;12(22):3647. doi: 10.3390/ma12223647 (PMC6887981; doi:10.3390/ma12223647)
Supplement: Supplementary file 1 [file materials-12-03647-s001.zip › supplementary/supplementary 7.docx]

Supplementary Materials

Mechanical Behaviour of Silicone Membranes Saturated with Short Strand, Loose Polyester Fibres for Prosthetic and Rehabilitative Surrogate Skin Applications

Richard Arm ^1,^*, Arash Shahidi ^1^ and Tilak Dias ^1^

Advanced Textiles Research Group, Flexural Composites Research Laboratory, School of Art and Design, Nottingham Trent University, Nottingham NG1 4GG, UK; arash.shahidi@ntu.ac.uk (A.S.); tilak.dias@ntu.ac.uk (T.D.)

***** Correspondence: richard.arm@ntu.ac.uk; Tel: +115-8488-6577.

Received: 4 October 2019; Accepted: 1 November 2019; Published: date

Coefficient Regression for PDMS 00-30 Fibre Influenced Hardness
