# Supplementary material for: Mechanical Behaviour of Silicone Membranes Saturated with Short Strand, Loose Polyester Fibres for Prosthetic and Rehabilitative Surrogate Skin Applications
Source: Materials (Basel). 2019 Nov 6;12(22):3647. doi: 10.3390/ma12223647 (PMC6887981; doi:10.3390/ma12223647)
Supplement: Supplementary file 1 [file materials-12-03647-s001.zip › supplementary/supplementary 8.docx]

Supplementary Materials

Mechanical Behaviour of Silicone Membranes Saturated with Short Strand, Loose Polyester Fibres for Prosthetic and Rehabilitative Surrogate Skin Applications

Richard Arm ^1,^*, Arash Shahidi ^1^ and Tilak Dias ^1^

Advanced Textiles Research Group, Flexural Composites Research Laboratory, School of Art and Design, Nottingham Trent University, Nottingham NG1 4GG, UK; arash.shahidi@ntu.ac.uk (A.S.); tilak.dias@ntu.ac.uk (T.D.)

***** Correspondence: richard.arm@ntu.ac.uk; Tel: +115-8488-6577.

Received: 4 October 2019; Accepted: 1 November 2019; Published: date

Uni-axial Tensile Test Results for PDMS A-10.

Zwick Roell Z2.5 tensile testing machine was used to study the physical behaviour of specimens during tensile testing. BS/ISO 37:2011 and ASTM D412 test standards were used to fix the test parameters to best suit the test specimen softness.

To prevent any slippage that can occur during the elongation of elastomeric membranes pneumatic grips with serrated jaw inserts were used [26] with grip to grip separation set to 25mm, preload of 0.5N and test speed at 50mm/min. In the interest of comparability, all graphs are presented here with maximum values set by the material (PDMS00-30@0% fibre) thresholds during tests.
